# The Chromosomal Passenger Complex and a Mitotic Kinesin Interact with the Tousled-Like Kinase in Trypanosomes to Regulate Mitosis and Cytokinesis

**DOI:** 10.1371/journal.pone.0003814

**Published:** 2008-11-26

**Authors:** Ziyin Li, Takashi Umeyama, Ching C. Wang

**Affiliations:** Department of Pharmaceutical Chemistry, University of California San Francisco, San Francisco, California, United States of America; Federal University of São Paulo, Brazil

## Abstract

Aurora B kinase plays essential roles in mitosis and cytokinesis in eukaryotes. In the procyclic form of *Trypanosoma brucei*, the Aurora B homolog TbAUK1 regulates mitosis and cytokinesis, phosphorylates the Tousled-like kinase TbTLK1, interacts with two mitotic kinesins TbKIN-A and TbKIN-B and forms a novel chromosomal passenger complex (CPC) with two novel proteins TbCPC1 and TbCPC2. Here we show with time-lapse video microscopy the time course of CPC trans-localization from the spindle midzone in late anaphase to the dorsal side of the cell where the anterior end of daughter cell is tethered, and followed by a glide toward the posterior end to divide the cell, representing a novel mode of cytokinesis in eukaryotes. The three subunits of CPC, TbKIN-B and TbTLK1 interact with one another suggesting a close association among the five proteins. An ablation of TbTLK1 inhibited the subsequent trans-localization of CPC and TbKIN-B, whereas a knockdown of CPC or TbKIN-B disrupted the spindle pole localization of TbTLK1 during mitosis. In the bloodstream form of *T. brucei*, the five proteins also play essential roles in chromosome segregation and cytokinesis and display subcellular localization patterns similar to that in the procyclic form. The CPC in bloodstream form also undergoes a trans-localization during cytokinesis similar to that in the procyclic form. All together, our results indicate that the five-protein complex CPC-TbTLK1-TbKIN-B plays key roles in regulating chromosome segregation in the early phase of mitosis and that the highly unusual mode of cytokinesis mediated by CPC occurs in both forms of trypanosomes.

## Introduction

*Trypanosoma brucei* is an ancient unicellular protozoan parasite that causes sleeping sickness in human and nagana in cattle in sub-Sahara Africa. The life cycle of *T. brucei* involves a cyclic transmission between the mammalian host and the insect vector tsetse fly with different developmental forms displaying distinct cellular morphology and biological features (for a review, see [1]). One of the differences between the bloodstream and insect (procyclic) forms lies in the regulation of cell division cycle (for a review, see [2]). Inhibition of mitosis in the latter does not totally block cytokinesis and results in anucleate daughter cells with single kinetoplasts (termed zoids) [3], [4]. In the bloodstream form, however, inhibition of mitosis prevents cytokinesis, but not additional rounds of G1 re-entry and organelle replication thus resulting in giant polyploid cells with multiple kinetoplasts, basal bodies and flagella [5]–[9]. It suggests that the procyclic form, but not the bloodstream form, lacks the checkpoint linking mitosis to cytokinesis, whereas the bloodstream form lacks a spindle assembly checkpoint. The molecular mechanisms behind these distinctions remain unclear at present and represent intriguing phenomena for further pursuit.

In an initial effort to dissect the molecular mechanisms of mitosis and cytokinesis in *T. brucei*, we characterized an Aurora-like kinase homolog TbAUK1, which is required for spindle formation, chromosome segregation and cytokinesis in both forms of *T. brucei* [5], [10]. TbAUK1 forms an unusual chromosomal passenger complex (CPC) in the procyclic form with two novel proteins TbCPC1 and TbCPC2 that exhibit no sequence homology to the well-characterized metazoan CPC components INCENP, Borealin/Dasra or Survivin [11]. Though this novel CPC displays a subcellular localization similar to that of the metazoan CPC by an association with the chromosomes before mitotic onset and a subsequent trans-localization to the spindle midzone in anaphase, it makes a most unusual move during telophase from the midzone across the nuclear envelope to a dorsal point on the cell where the anterior end of daughter cell is tethered [11]. The CPC then makes an apparent downward movement toward the posterior end and separates the daughter from the mother. This is an unusual way of forming a cleavage furrow and a unique mode of cytokinesis that has not yet been observed in any other eukaryote. This novel finding should require more supporting evidence for further confirmation and an examination in the bloodstream form for its pattern of cytokinesis.

TbAUK1 also interacts with two novel kinesin-like proteins TbKIN-A and TbKIN-B, both of which are required for the subsequent translocation of CPC from the chromosomes to the spindle midzone and to the unusual cleavage furrow, though they do not co-migrate with the CPC themselves in the procyclic form [11]. We have also previously identified a substrate of TbAUK1 in trypanosomes [12]; a homolog of Tousled-like kinase (TbTLK1) that interacts with and becomes phosphorylated by TbAUK1. It plays an essential function in spindle assembly and chromosome segregation and localizes to the spindle poles during mitosis [12]. Thus, the CPC, the two mitotic kinesin homologs and TbTLK1 could constitute a six protein complex in the trypanosomes regulating the early events in mitosis.

In this report, we used time-lapse video microscopy to further confirm and define the novel mode of cytokinesis in the procyclic form of *T. brucei*. The same unique pattern of cytokinesis was also demonstrated in the bloodstream form thus establishing *T. brucei* as a useful model for further research of cytokinesis. We also indicated that TbTLK1 indeed interacts with TbCPC1, TbCPC2, TbKIN-B as well as TbAUK1, but not with TbKIN-A. A TbTLK1 knockdown abolished the trans-localizations of CPC and TbKIN-B from nucleus to central spindle/spindle midzone, and a depletion of the three CPC components or TbKIN-B disrupted the localization of TbTLK1 to the spindle poles. The same five proteins are also expressed in the bloodstream form and have similar patterns of trans-localizations and functions. Thus, in spite of the many distinctions in cell cycle regulation between the two forms, the critical functions of the five-protein complex, the unusual cleavage furrow formation and the unique mode of cytokinesis mediated by CPC remain the same.

## Results

### Time-lapse video fluorescence microscopic analysis of TbCPC1-EYFP trans-localization during mitosis and cytokinesis in the procyclic form

**Figure 1:**
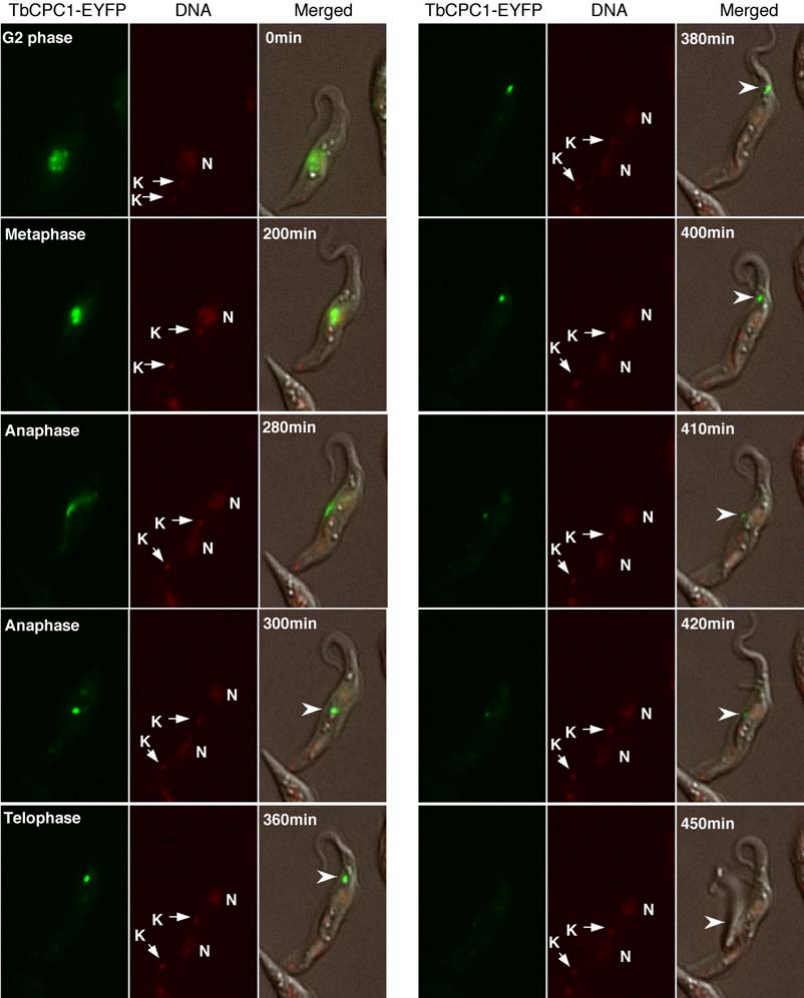
Translocalization of TbCPC1-EYFP in live cell imaged by video fluorescence microscopy. Procyclic cell stably expressing the enhanced yellow fluorescent protein (EYFP) tagged TbCPC1 was imaged during mitosis-cytokinesis. DNA was stained with Hoechst DNA dye, and the fluorescence and phase images were merged. Selected images at various time intervals are shown to illustrate the trans-localization of TbCPC1-EYFP (arrowheads) from nucleus to spindle midzone and then to dorsal side toward the anterior end and finally to the posterior end of the cell. Nucleus (N) and kinetoplast DNA (K) are indicated. The complete image sequence is available as a video in the Supplementary file Movie S1.

To confirm the unique pattern of trans-localization of CPC during cytokinesis, video fluorescence microscopy was used to follow the movement of TbCPC1 tagged with the enhanced yellow fluorescent protein (EYFP) in procyclic form cells. To this end, a *TbCPC1-EYFP* fusion gene was integrated into one allele of the *TbCPC1* loci in the genome and correct *in situ* tagging of this allele was confirmed by PCR. The cells expressing TbCPC1-EYFP were then plated on the surface of 1% agarose gel prepared in cultivation medium without phenol red and air dried briefly to limit cell motility. Time-lapse images of individual cells were acquired with a 6D High Throughput Microscope at 5 to 8 multi-points for 12 hrs with 10 min intervals. Selected images at different time intervals were shown in Figure 1 and the complete video can be found in the Supplementary file Movie S1. TbCPC1-EYFP was initially identified in a punctate distribution in the nucleus during G2 phase, presumably in association with the chromosomal kinetochores (Fig. 1, 0 min). It was then concentrated on the metaphase plane (Fig. 1, 200 min) and focused into a bright dot in the spindle midzone in late anaphase (Fig. 1, 300 min). The dot then started to move toward the dorsal side of the cell where the anterior end of the daughter cell was tethered (Fig. 1, 360 min and 380 min). Subsequently, the fluorescent dot migrated from the anterior end toward the posterior end along the presumed cleavage furrow to divide the cell into two (Fig. 1, 400 to 450 min). This dynamic localization pattern of TbCPC1-EYFP confirmed the conclusion derived from the previous data based on immunofluorescence assays [11] and further illustrated the most unusual pattern of cytokinesis in *T. brucei*. However, since the cells were placed in semi-solid agarose to minimize the motility, they might not be under the most optimal physiological conditions for division. The time period thus observed for its completion could have been artificially prolonged. Further readjustments of experimental conditions will be necessary for a more accurate assessment of the time course of cytokinesis in procyclic *T. brucei* **.**

### TbTLK1 interacts with TbCPC1, TbCPC2, TbAUK1 and TbKIN-B in yeast and in *T. brucei*

**Figure 2:**
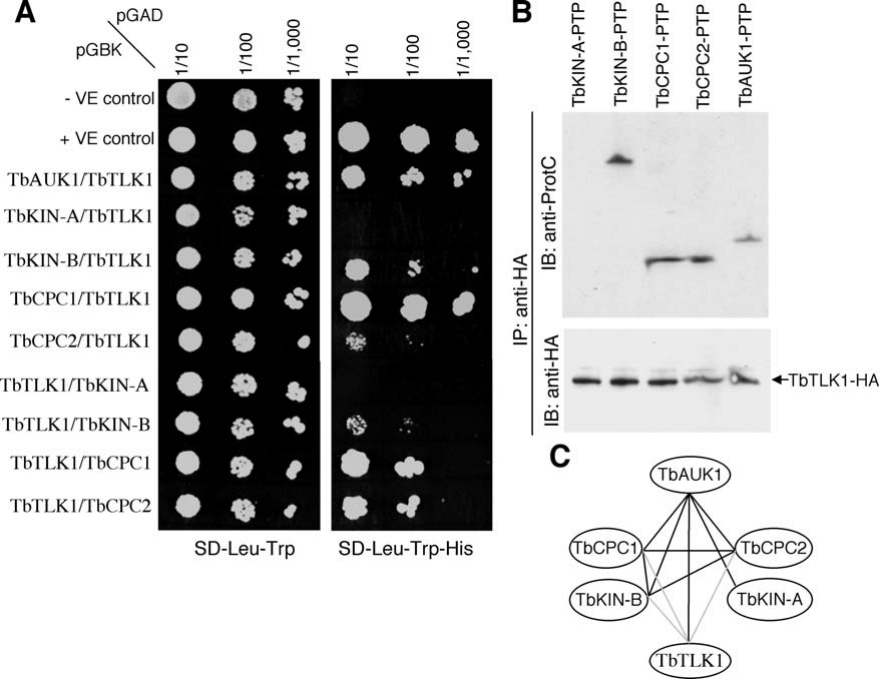
Interactions among TbTLK1 and TbKIN-B, TbCPC1, TbCPC2 and TbAUK1. (A). Yeast two-hybrid assay. Full-length TbTLK1, TbKIN-A, TbKIN-B, TbCPC1, TbCPC2 and TbAUK1 were each cloned into the pGADT7 vector for expression of proteins fused to the Gal4 activation domain (prey) or into the pGBKT7 vector for expression of proteins fused to the Gal4 binding domain (bait), transformed to yeast strains AH109 and Y187, respectively. Each mated strain was then spotted onto SD-Leu-Trp and SD-His-Leu-Trp plates; with the latter selecting for interacting bait and prey proteins. (B). Co-immunoprecipitation testing potential interactions between TbTLK1-HA and TbKIN-A, TbKIN-B, TbCPC1, TbCPC2 or TbAUK1, each of which was tagged with a PTP epitope and expressed in trypanosome to the endogenous level. (C). The interaction map among the six proteins summarized from yeast two-hybrid, GST pull down and co-immunoprecipitation assays in our previous studies (black lines; [11], [12]) and the current study (gray lines).

Since TbTLK1 is known to bind to TbAUK1 *in vitro* and *in vivo* in trypanosomes [12], we tested whether TbTLK1 also interacts with the other proteins known to form complexes with TbAUK1. To this end, we first employed the yeast two-hybrid assays by pairing TbTLK1 with TbKIN-A, TbKIN-B, TbCPC1, TbCPC2 and TbAUK1 for potential interactions. We found that when TbTLK1 was fused to the Gal4 activation domain (pGADT7), it binds to TbCPC1 and TbAUK1 with strong affinity and to TbCPC2 and TbKIN-B with somewhat weaker affinity, but it does not bind to TbKIN-A (Fig. 2A). Conversely, when TbTLK1 was fused to the Gal4 binding domain (pGBKT7), it binds to TbCPC1 and TbCPC2 with high affinity and to TbKIN-B with lower affinity, but it does not bind to TbKIN-A or TbAUK1 (Fig. 2A). The negative outcome between TbTLK1 and TbAUK1 in the latter test could be attributed to unfavorable conformational change(s) in either protein, since interaction between the two proteins has been well established in our previous studies [12].

To confirm in trypanosomes the observed protein-protein interactions (Fig. 2A), we employed the co-immunoprecipitation experiment. TbTLK1 tagged with a triple HA epitope was expressed to the presumed endogenous level through homologous recombination in transfected procyclic cells. TbKIN-A, TbKIN-B, TbCPC1, TbCPC2, or TbAUK1 were each tagged with a PTP (proteinA/tobacco etch virus protease site/proteinC) epitope [13] and expressed also to the putative endogenous levels through homologous recombination in the TbTLK1-3HA expressing cells. Immunoprecipitation was performed with anti-HA pAb to pull down TbTLK1-3HA. The immunoprecipitates were then immuno-blotted with anti-ProtC mAb for PTP-tagged proteins. As previously reported [12], TbTLK1 was able to pull down TbAUK1 (Fig. 2B). In addition, it was found also capable of pulling down TbKIN-B, TbCPC1 and TbCPC2 but not TbKIN-A (Fig. 2B). These results, indicating that TbTLK1 interacts with all three subunits of the CPC and one of the two essential kinesin-like proteins TbKIN-B in trypanosomes, are in agreement with the results from the yeast two-hybrid assay and suggest a complex of the five proteins (Fig. 2A). TbKIN-A does not interact with TbCPC1, TbCPC2, TbKIN-B or TbTLK1 (Fig. 2C). It interacts with only TbAUK1 and may not be a component of the postulated complex [11] (see Discussion).

### RNAi knockdown of TbTLK1 abolishes the trans-localization of TbKIN-B and the CPC from nucleus to central spindle

**Figure 3:**
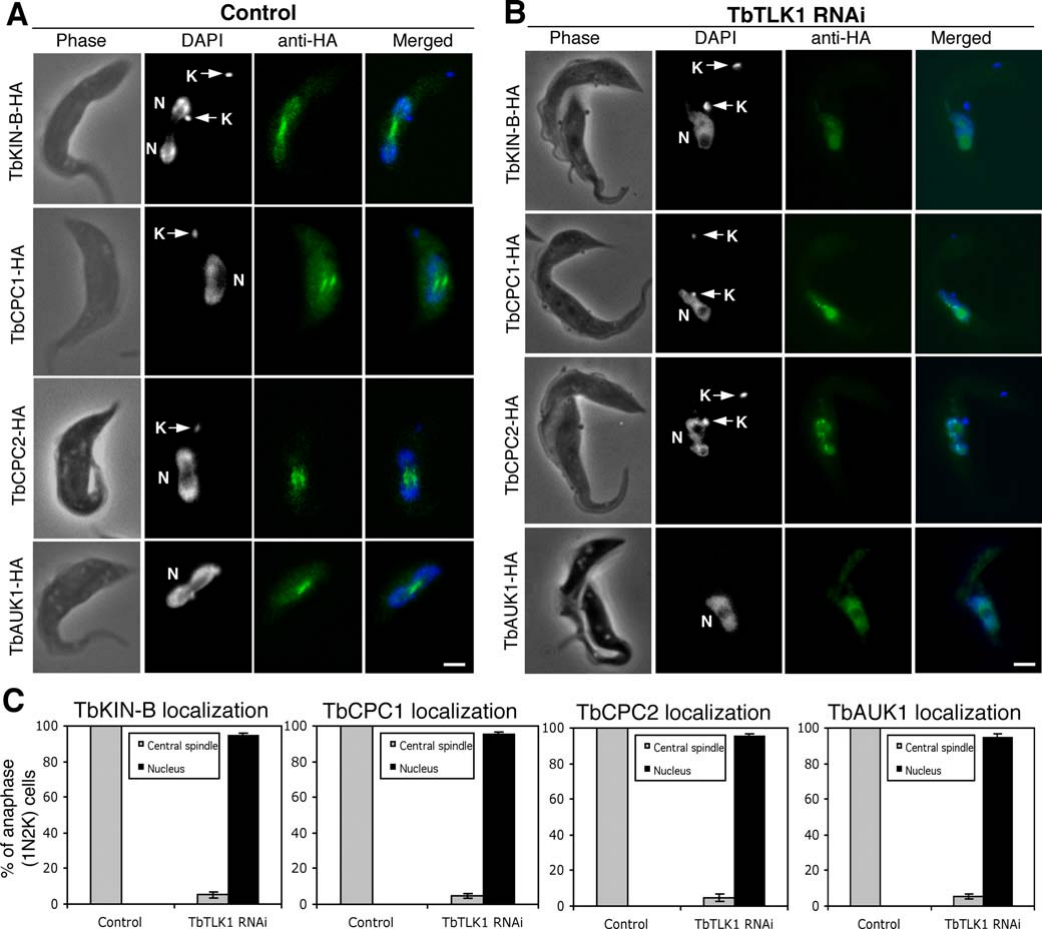
Effects of TbTLK1 knockdown on subcellular localizations of TbKIN-B, TbCPC1, TbCPC2 and TbAUK1. (A). TbKIN-B-3HA, TbCPC1-3HA, TbCPC2-3HA and TbAUK1-3HA expressed to the endogenous levels in un-induced RNAi cells were detected with FITC-conjugated anti-HA mAb. (B). Localization of the 3HA-tagged TbKIN-B, TbCPC1, TbCPC2 and TbAUK1-3HA after a 24 hr RNAi of TbTLK1. All stained cells were of the 1N2K type (N, nucleus; K, kinetoplast) in the anaphase stage of cell cycle. (C). Percentages of 1N2K cells with TbKIN-B-3HA, TbCPC1-3HA, TbCPC2-3HA and TbAUK1-3HA localized to the central spindle. Data are presented as the mean percent ±S.D. of ∼300 cells counted from three independent experiments. Bars: 2 µm.

We next investigated the potential effect of TbTLK1 knockdown on the subcellular distribution of TbKIN-B and the three subunits of CPC in the procyclic cells. Each of the four proteins was tagged with a triple HA epitope at the C-terminus and expressed to the presumed endogenous levels via homologous recombination in the TbTLK1 RNAi cells. Knockdown of TbTLK1 mRNA was confirmed by RT-PCR, and expression of each of the 3HA-tagged proteins was demonstrated by Western blot using anti-HA antibody (data not shown). Localizations of the four proteins before and after TbTLK1 RNAi were then examined by immunofluorescence. In the control cells, TbKIN-B localized to the nucleus and concentrated on the central spindle during anaphase, whereas the three subunits of CPC were focused on the central spindle during anaphase (Fig. 3A) similar to the previous observations [11]. When TbTLK1 was knocked down, however, TbKIN-B and the three components of CPC were found all localized to the nucleus in a diffused manner among more than 95% of the cells with one nucleus and two kinetoplasts (1N2K), representing cells blocked from chromosome segregation and cytokinesis (Fig. 3B and 3C). Depletion of TbTLK1 thus abolished the characteristic patterns of trans-localization of TbKIN-B and CPC from nucleus to central spindle during mitosis.

### RNAi knockdown of the three CPC components or TbKIN-B disrupts the localization of TbTLK1 to the spindle poles

**Figure 4:**
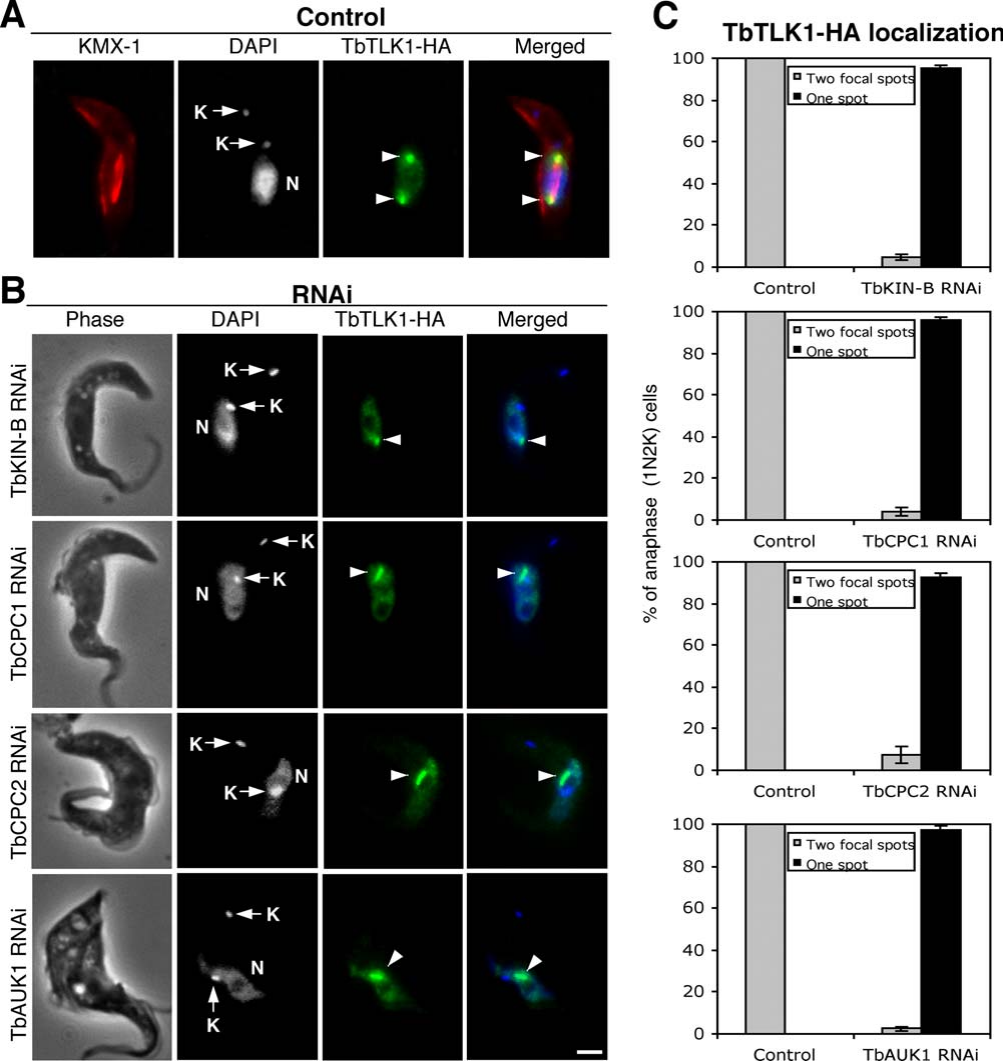
Effects of TbKIN-B, TbCPC1, TbCPC2 and TbAUK1 knockdowns on subcellular localization of TbTLK1. (A). The TbTLK1-3HA expressed to the endogenous level in wild-type cells was detected with FITC-conjugated anti-HA mAb. Spindle structure is detected by immunostaining with KMX-1 antibody and Cy3-conjugated secondary antibody. Arrowheads point to the two focal spots at the spindle poles. N, nucleus; K, kinetoplast. (B). TbTLK1-3HA expressed in cells harboring the TbKIN-B, TbCPC1, TbCPC2 or TbAUK1 RNAi construct was detected with FITC-conjugated anti-HA mAb after a 48 hr RNAi induction. All stained cells were of the 1N2K type. Arrowheads point to the concentrated regions of TbTLK-3HA in the nucleus. (C). Percentages of 1N2K cells with one concentrated TbTLK-3HA region in the nucleus. Data are presented as the mean percent ±S.D. of ∼300 cells counted from three independent experiments. Bar: 2 µm.

TbTLK1 tagged with a triple HA epitope at its C-terminus was expressed to the presumed endogenous level via homologous recombination in the four clonal RNAi cell lines depleted of TbKIN-B or the three CPC subunits. Knockdowns of individual genes were verified by RT-PCR (data not shown). Localization of TbTLK1-3HA was then examined with immunofluorescence. In the wild-type cells, TbTLK1-3HA localized to the nucleus and forms two bright focal points in the nucleus during metaphase and anaphase corresponding to the two spindle poles (Fig. 4A, arrowheads). When TbKIN-B and the three CPC components were each knocked down, however, TbTLK1-3HA remained in the nucleus of 1N2K cells (Fig. 4B), but did not move to the two spindle poles. Instead, there appeared to be areas of concentrated TbTLK1-3HA inside the nucleus among most of the 1N2K cells (Fig. 4B and 4C, arrowheads). The nature of these concentrated areas is not clear at present. But the localization of TbTLK1 to the two spindle poles was clearly disrupted.

### TbCPC1, TbCPC2 and TbAUK1 display subcellular localization patterns typical of the chromosomal passenger complex in the bloodstream form of *T. brucei*

**Figure 5:**
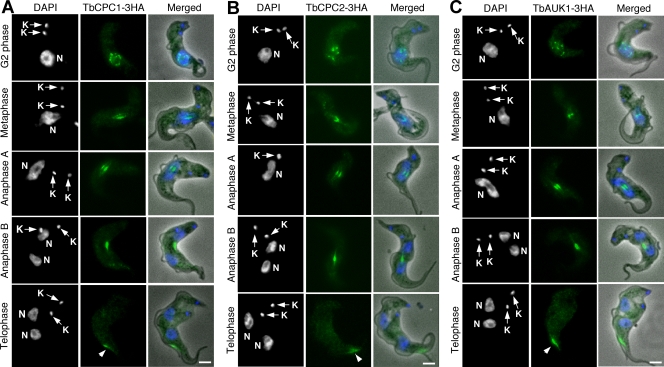
Subcellular localizations of TbCPC1, TbCPC2 and TbAUK1 in the bloodstream trypanosomes. Cells expressing 3HA-tagged TbCPC1 (A), TbCPC2 (B) and TbAUK1 (C) proteins to the endogenous levels were fixed, stained with FITC-conjugated anti-HA antibody, and counterstained with DAPI for nuclear (N) and kinetoplast (K). The cell populations consisted of G2 phase (9–11% of the population); metaphase (4–6%); anaphase A (5–7%); anaphase B (7–9%); telophase (5–8%). About 600 cells from each sample were examined and essentially all the cells were stained. The G1-phase (51–58%) and S-phase (8–12%) cells do not express these three HA-tagged proteins. Arrowheads point to the localization of CPC to the dorsal side of cell body. Bars: 2 µm.

It is not known whether TbCPC1, TbCPC2 and TbAUK1 form also a CPC in the bloodstream form. By tagging each encoding gene with a triple HA epitope in the bloodstream cells and expressing them to the presumed endogenous level followed by immunofluorescence microscopy, we found the three proteins closely associated with the nucleus in G2 phase, trans-localized to the central spindle in metaphase and anaphase A, and then concentrated in the spindle midzone in anaphase B (Fig. 5). In telophase, they were trans-localized in the form of a bar-shaped entity to the dorsal side of the cell body near the anterior end, where the anterior ends of both mother and daughter cells are known to tether (Fig. 5, arrowheads). They then made an apparent move toward the posterior end during cytokinesis. This pattern of trans-localization is very similar to that observed in the procyclic form [11].

These data led us to the conclusion that TbAUK1, TbCPC1 and TbCPC2 form the same CPC and trans-localize in the same unique pattern at the end of mitosis in both forms of *T. brucei* (see Discussion).

### Subcellular localizations of TbKIN-A, TbKIN-B and TbTLK1 in the bloodstream form of *T. brucei*

**Figure 6:**
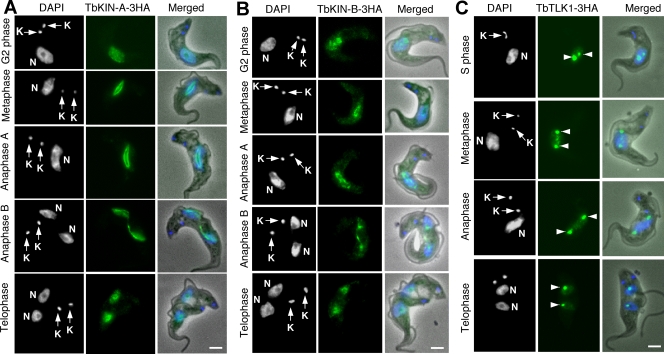
Subcellular localizations of TbKIN-A, TbKIN-B and TbTLK1 in the bloodstream trypanosomes. Cells expressing 3HA-tagged TbKIN-A (A), TbKIN-B (B), and TbTLK1 (C) proteins to the endogenous levels were fixed, stained with FITC-conjugated anti-HA antibody, and counterstained with DAPI for nucleus (N) and kinetoplast (K). Percentages of cells at different cell cycle stages were determined from samples of about 500 cells. For cells expressing TbKINA-3HA or TbKIN-B-3HA, the distributions were as the following, respectively; G2 phase (10%, 9%); metaphase (8%, 7%); anaphase A (7%, 6%); anaphase B (8%, 10%); telophase (6%, 7%), whereas the G1 phase (53%, 54%) and S-phase (8%, 7%) cells did not have detectable TbKIN-A-3HA or TbKIN-B-3HA. The TbTLK-3HA cells had S phase (12%); metaphase (8%); anaphase (11%) and telophase (6%) cells expressing the protein but the G1-phase cells (63%) did not express TbTLK1-3HA. The arrowheads point to the two focal spots at the likely spindle poles. Bars: 2 µm.

TbKIN-A, TbKIN-B and TbTLK1, each tagged at the C-terminus with a triple HA epitope and expressed to the presumed endogenous level through homologous recombination in the bloodstream form, were examined with immunofluorescence microscopy. The results (Fig. 6) indicated that the localization patterns of TbKIN-A, TbKIN-B and TbTLK1 in the bloodstream form are very much like that in the procyclic form 11, 12, suggesting similar roles of these proteins in both forms.

### RNAi knockdown of the five TbAUK1-associated proteins in the bloodstream form results in defects in chromosome segregation and cytokinesis

**Figure 7:**
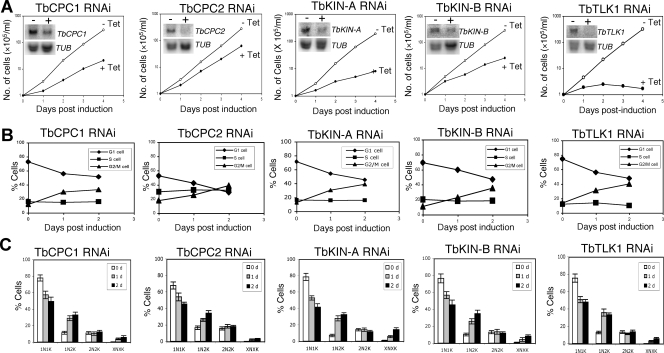
RNAi silencing of TbCPC1, TbCPC2, TbKIN-A, TbKIN-B, and TbTLK1 in the bloodstream form of *T. brucei*. (A). Clonal cell lines harboring the RNAi constructs were cultivated with (+Tet) or without (−Tet) tetracycline induction and monitored for cell growth. Northern was performed to monitor the levels of mRNA in cells before (−) and after (+) RNAi for 2 days. α-Tubulin (*TUB*) mRNA was monitored as a control. (B). Flow cytometry analysis of the DNA contents in RNAi cells. (C) RNAi cells after tetracycline induction for 0, 1 and 2 days were stained with DAPI and tabulated for numbers of nuclei (N) and kinetoplasts (K) in each cell. Data are presented as the mean percent ±S.D. of ∼200 cells counted from three independent experiments. The experiment was repeated with three independent RNAi clones, and the results thus obtained were very similar to one another.

RNAi of the five TbAUK1-associated proteins in the bloodstream cells was performed and clonal cell lines were treated with tetracycline for potential phenotypes. Northern blots showed significant reduction of individual mRNAs after RNAi induction for 2 days (Fig. 7A, insets). Appreciable growth inhibition was observed in all five RNAi cell lines with the TbTLK1 knockdown showing the severest effect leading to eventual cell death within five days (Fig. 7A). Each of the five genes could thus play an essential role in bloodstream cell division.

To examine the potential defects in cell cycle progression in the five RNAi cell lines, flow cytometry was performed, which showed, in every case, an accumulation of G2/M cells (4C DNA content) and a decrease of G1 cells (2C DNA content) (Fig. 7B) similar to that observed in the procyclic form [11], [12]. These genes are thus obviously required for mitotic progression in the bloodstream cells.

**Figure 8:**
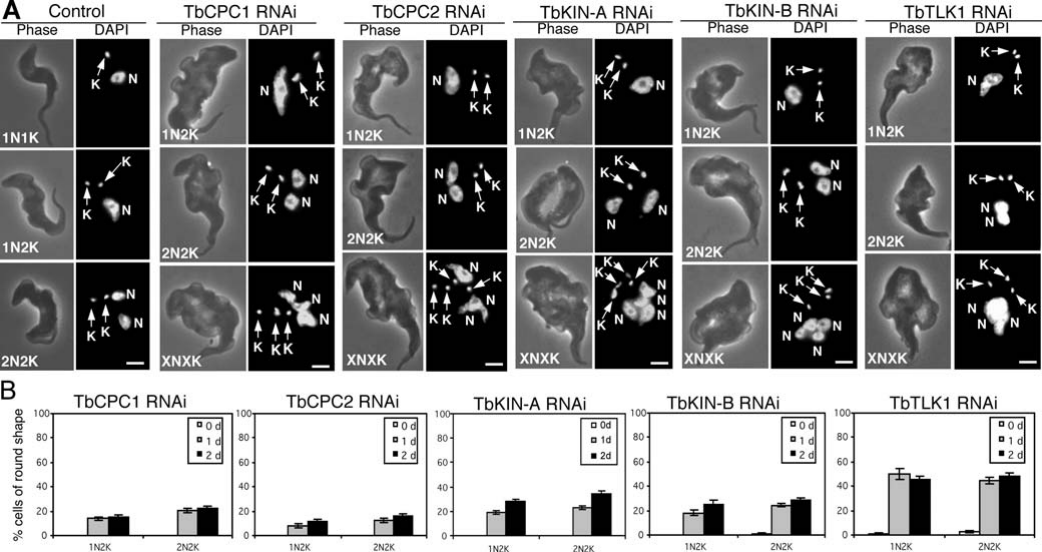
Morphology of the control and knockdown bloodstream cells after RNAi induction. (A). Control cells and cells after RNAi induction for 48 hrs were fixed with 4% paraformaldehyde, stained with DAPI for nucleus (N) and kinetoplast (K), and examined under fluorescence microscope. Bars: 2 µm. (B). Percentages of round-shaped cells after RNAi silencing of TbCPC1, TbCPC2, TbKIN-A, TbKIN-B and TbTLK1 for 24 and 48 hrs. About 200 cells were counted in each sample and the data represent three independent experiments.

The RNAi cells after a two-day induction were then stained with DAPI for numbers of nuclei and kinetoplasts in each cell. Again, the outcome turned out to be quite similar among the five cell lines and mimicking those results from the procyclic form [11], [12]. The number of cells with one nucleus and one kintoplast (1N1K) gradually decreased, and the number of 1N2K cells increased significantly (Fig. 7C), which showed mostly an enlarged and elongated nucleus (Fig. 8A). This is similar to the outcome from knocking down TbAUK1 from the bloodstream form [5], indicating a common defect in chromosome segregation and an arrest of cytokinesis. Furthermore, there was a slight increase in the number of cells with a big nuclear aggregate and multiple kintoplasts (XNXK) among ∼5% of the five RNAi cell lines (Figs. 7C and 8A), suggesting that additional rounds of nuclear DNA and kinetoplast replication occurred in these cells while cytokinesis is blocked. Such XNXK cells, also previously observed in the bloodstream cells depleted of TbAUK1 [5], representing one of the main distinctions in cell cycle regulation between the bloodstream and procyclic forms (see Discussion).

### RNAi knockdown of the five TbAUK1-associated proteins in the bloodstream form leads to distorted cell morphology

**Figure 9:**
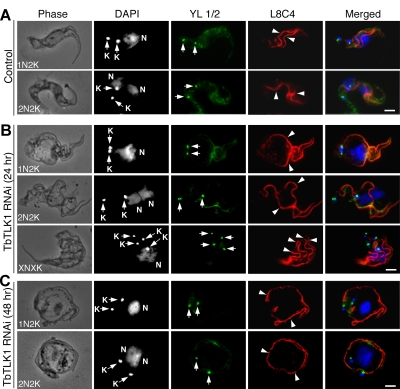
Effect of TbTLK1 knockdown on replication and segregation of the kinetoplasts, basal bodies and flagella in bloodstream cells. Control and RNAi cells were fixed in cold methanol, immunostained with YL1/2 antibody for basal bodies (arrows) and L8C4 antibody for flagella (arrowheads), and counterstained with DAPI for kinetoplast (K) and nucleus (N). (A). Control cells; (B). TbTLK1 RNAi cells with rounded posterior end after 24 hrs; (C). TbTLK1 RNAi cells of round shape after 48 hrs. Bars: 2 µm.

An intriguing phenotype shared by depleting the five proteins from the bloodstream form was the grossly distorted cell morphology. After RNAi for two days, the 1N2K and 2N2K cells gradually became rounded up at the posterior end two to approximately threefold wider than that of the wild type cells (Fig. 8A). In some cases, the cells became completely spherical in shape (see Fig. 9C below). About 50% of the cells became rounded after RNAi silencing of TbTLK1, whereas about 15–25% became rounded at the posterior end after depletion of TbCPC1, TbCPC2, TbKIN-A or TbKIN-B for 1 to 2 days (Fig. 8B). This morphological change is similar to that of TbAUK1-deficient bloodstream cells [5].

We further characterized the spherical 1N2K and 2N2K cells from a TbTLK1 knockdown by immunostaining the flagellum with L8C4 and the basal body with YL1/2 antibodies. All the cells possessed two full-length flagella that were widely separated from each other albeit still attached to the cell body, which was in striking contrast to the control cells in which the two flagella attached in close parallels on the dorsal side of the cell body (compare Fig. 9A with 9B and 9C, arrowheads). There were also two segregated basal bodies (Figs. 9B and 9C, arrows), indicating that their replication and segregation were not affected by the TbTLK1 knockdown. In the XNXK cells, multiple basal bodies and flagella were seen (Fig. 9B), suggesting additional rounds of basal body duplication and flagellum synthesis in the cells blocked from cytokinesis, a phenotype similar to that from knocking down TbAUK1 [5].

## Discussion

Originally discovered in plant [14], the TLKs comprise an evolutionarily conserved family of serine/threonine protein kinases among multi-cellular eukaryotes. TLK-1 functions as a substrate activator of the Aurora B in *Caenorhabditis elegans* [15], and phosphorylates the chromatin assembly factor Asf1 to regulate DNA replication in human, *Drosophila* and plant [16]–[18]. Close homologs of TLK are absent from unicellular eukaryotes including *Saccharomyces cerevisiae*, which led to the assumption that TLKs are present only in metazoans and likely function in some fundamental aspects of development common to plants and animals [18], [19]. Since our identification of a functioning TLK homolog in *T. brucei* [12], which interacts with TbAUK1 and TbAsf1 in regulating chromosome segregation and DNA replication, the mechanisms by which TLK regulates cell division are apparently conserved among the eukaryotic microbes as well. But TbTLK1 plays also an additional function in *T. brucei* by localizing to the spindle poles and regulating the spindle assembly, which has not been observed previously in metazoans [12].

We showed in this report that TbTLK1 interacts with all three subunits of the CPC in trypanosomes (Fig. 2) and is required for the trans-localization of CPC to the central spindle and the spindle midzone (Fig. 3). Since TbCPC1 and TbCPC2 exhibit no sequence similarity to those known CPC components in metazoans [11], it would be interesting to investigate if the TLKs in metazoans interact also with INCENP, Borealin/Dasra or Survivin and whether the TLKs are required for CPC localization to the spindle midzone in metazoans as well. Furthermore, we found that each of the three CPC subunits in *T. brucei* is also essential for the focalized localization of TbTLK1 to the spindle poles (Fig. 4), suggesting that TbTLK1 and CPC may be interdependent for their subcellular localization.

In addition, we demonstrated that TbKIN-B interacts with TbTLK1 and the three components in CPC (Fig. 2). Kinesins constitute a super-family of microtubule-based motor proteins that perform diverse cellular functions (for a review, see [20]), and several kinesins are required for mitosis and cytokinesis [21]–[24]. TbKIN-B lacks several well-conserved and essential residues in the motor domain such as the lysine residue in the NTP-binding motif and the SSRSH motif [11]. It bears only ∼15% identity to BimC, MKLP1, CENP-E and MCAK, raising the possibility that it may not be a functioning kinesin. Knockdown of TbTLK1 disrupted the focal concentration of TbKIN-B at the central spindle/spindle midzone, whereas silencing of TbKIN-B abolished the focal localization of TbTLK1 to the two spindle poles (Figs. 3 and 4). This is the first time, to our knowledge, that a TLK is found interacting with a kinesin homolog, and both proteins are mutually dependent for their respective subcellular localizations. It is also the first indication that TLK, a kinesin homolog and a CPC form a complex prior to mitosis. The five proteins were found localized in the nucleus of the G2 phase cells [11], [12], which could be when the five-protein complex was formed and demonstrated in the immunoprecipitation experiments.

TbKIN-A, a novel kinesin protein that cannot be classified into any known kinesin group, has the typical N-terminal kinesin motor domain and several coiled-coil motifs at the C-terminus [11]. Its motor domain is ∼30% identical to that of BimC, MKLP1, CENP-E and MCAK, and could thus possess the function of a kinesin. Its confinement within the nucleus throughout mitosis [11] and its lack of interaction with TbTLK1, TbCPC1, TbCPC2 or TbKIN-B suggests that it may be primarily associated with the chromosomes and interact with TbAUK1 only during the early phase of mitosis.

The five-protein complex has apparently similar patterns of trans-localizations and functions in both procyclic and bloodstream forms. However, an RNAi knockdown of TbTLK1, TbKIN-B, TbCPC1 and TbCPC2 in the bloodstream form produced also polyploid cells with multiple kinetoplasts, basal bodies and flagella (Figs. 7C, 8A and 9B), whereas a similar knockdown in the procyclic form led to cells with single nucleus approximately doubled in size and two segregated kinetoplasts, basal bodies and flagella [11], [12]. This distinction plus a previously identified difference that the procyclic form may not possess the mitosis to cytokinesis checkpoint while the bloodstream form may have it could constitute the major distinctions between the two forms ready for further investigation.

Another intriguing observation from the present study is the grossly distorted cell morphology in the bloodstream form depleted of the five components of the complex (Figs. 8 and 9), which is similar to the morphology of TbAUK1-deficient bloodstream form [5]. But similar morphology change was not observed in the bloodstream form depleted of a mitotic cyclin CycB2 (unpublished data) or a mitotic cdc2-related kinase CRK3 [6], both of which are essential for G2/M transition in trypanosomes. The results thus argue for an essential role of the CPC and its associated proteins in maintaining cell morphology in the bloodstream form. Our previous results have shown that excessive amount of microtubules accumulated in the posterior end of the TbAUK1-deficient bloodstream cells [5]. Similarly, inhibition of the Aurora B activity in *Xenopus* eggs perturbs mitotic microtubule dynamics with an elaboration of astral arrays [25]. It is thus likely that the CPC and its associated proteins may exert a regulatory function on microtubule dynamics in the bloodstream cells.

Finally, the unusual mode of cytokinesis initially observed in the procyclic form in immunofluorescence assays [11] was further examined in living procyclic cells expressing TbCPC1-EYFP. Using time-lapse video microscopy, we followed the trans-localization of TbCPC1-EYFP and were able to fully confirm our previous conclusion [11]. Starting from the early G2 phase, the protein had a punctate distribution in the nucleus indicating probable association with the chromosomal kinetochores. It then moved to the metaphase spindle and focused at the midzone of spindle in late anaphase before a dramatic trans-localization to the dorsal side of the mother cell where the anterior end of daughter cell was tethered. The protein spot made a move toward the anterior end and then migrated rapidly toward the posterior end resulting in two separated cells (Fig. 1 and Movie S1). The same pattern of cytokinesis was also observed in the bloodstream form with an immunofluorescence assay in the current study (Fig. 5). We consider these observations an important finding, because no other eukaryote has been found to have a similar mode of cytokinesis. It has been known that the contractile actomyosin ring that constitutes the cleavage furrow in other eukaryotes is not present in the trypanosomes [26]. Classical microscopic evidence indicated that the trypanosomes divide from the anterior end, split the mother from the daughter cell longitudinally and eventually separate them at the posterior point [11]. We believe that the present work and the previous study [11] have consolidated this novel discovery and provided a molecular basis for this most unusual mode of cytokinesis. The CPC in both forms of trypanosomes would trans-localize in late anaphase from the spindle midzone across the nuclear envelope to the anterior tip of daughter cell to initiate cytokinesis. It differs significantly from the cytokinesis in metazoans, in which the CPC remains in the midzone and generates a gradient of phosphorylated proteins in late anaphase [27]. This gradient then most likely directs and controls the contraction of cleavage furrow to close onto the midzone to accomplish cell division. Our discovery of a completely new mechanism of cytokinesis in an ancient eukaryote may provide an exciting opportunity for studying the evolution of cytokinesis in eukaryotes.

## Materials and Methods

### Live cell imaging

TbCPC1 was cloned into the pC-EYFP-Neo vector and transfected into the procyclic form strain 427 cell line to express TbCPC1-EYFP to the presumed endogenous level [28]. Correct *in situ* tagging of one of the two *TbCPC1* alleles was confirmed by PCR and subsequent sequencing. Stable transfectants were selected under 40 µg/ml G418. To follow the trans-localization of TbCPC1-EYFP with time-lapse imaging, a melted 1% low melting point agarose mixture in 1.2 ml of SDM-medium without phenol red and 5 µl Hoechst 33342 solution (1 mg/ml, Invitrogen) was poured onto the center of a slide-glass and covered with another slide-glass. The top side-glass was then removed from the agarose pad 10–15 min later and a slurry of the transfected cells was poured onto the pad and covered with a cover-slip, which was sealed using a 1∶1∶1 mixture of vaseline, lanolin and paraffin. Time-lapse images of individual cells were acquired with a 6D High Throughput Microscope at the Nikon Imaging Center at UCSF (http://nic.ucsf.edu/6D.html). The images were taken at 5 to 8 multi-points with a fixed time interval (10 min). An auto-focusing program using DIC images was installed that produces images with Z-stacks at –1, 0 and +1 µm from the auto-focused plane.

### Trypanosome Cell Cultures and Inducible RNA Interference

The bloodstream form of *T. brucei* strain 90-13 [29] was grown at 37°C with 5% CO_2_ supplied in the HMI-9 medium containing 10% fetal bovine serum and 10% serum plus (JRH) and 2.5 µg/ml G418 and 5 µg/ml hygromycin B. The procyclic form of *T. brucei* cells harboring RNAi constructs [11], [12] were cultivated at 26°C in Cunningham's medium supplemented with 10% fetal bovine serum (Atlanta Biological) and 15 µg/ml G418, 50 µg/ml hygromycin B and 2.5 µg/ml phleomycin. Cells were routinely diluted with the fresh medium once the density reached 5×10^6^ cells/ml.

The RNAi constructs were linearized by *Not*I digestion so that they could be integrated into the rDNA spacer region of the *T. brucei* chromosomes. Transfection of both forms of *T. brucei* by electroporation was performed essentially according to our previously described procedures [5], [30]. The transfectants were selected under 2.5 µg/ml phleomycin and cloned by plating on the agarose plate [31]. RNAi was induced by adding 1.0 µg/ml tetracycline to switch on the opposing T7 promoters. Cell numbers at different time intervals were counted under a microscope using a hemocytometer.

### Northern Blot and RT-PCR

Total RNA was blotted onto nitrocellulose membrane. Northern hybridization was carried out overnight at 42°C in 50% formamide, 6× SSC, 0.5% SDS, 5× Denhardt's solution with 0.1 mg/ml salmon sperm DNA. The same blot was probed with α-tubulin fragment for equal loading of RNA samples.

Total RNA was treated with DNase I to remove contaminating DNA and used to generate the first-strand cDNAs. PCR was performed using 200 ng first-strand cDNAs and gene-specific primers that were different from the primer pair used in generating the RNAi constructs. The PCR cycling program was set for 28 cycles at 94°C for 15 s, 55°C for 30 s, and 72°C for 30 s followed by a final elongation time at 72°C for 5 min.

### Flow Cytometry Analysis

The fluorescence-activated cell sorting scan (FACScan) analysis of propidium iodide-stained trypanosome cells was carried out as previously described [30]. Briefly, *T. brucei* cells were fixed in ethanol and incubated with DNase-free RNase (10 µg/ml) and propidium iodide (20 µg/ml) before flow cytometry analysis. The DNA content of propidium iodide-stained cells was analyzed with a FACScan analytical flow cytometer (BD Biosciences). Percentages of cells in each phase of the cell cycle (G1, S, and G2/M) were determined with the ModFit LT V3.0 software (BD Biosciences).

### Epitope Tagging of Endogenously Expressed Proteins in T. brucei

TbTLK1, TbKIN-A, TbKIN-B, TbCPC1, TbCPC2 and TbAUK1 were each cloned into the pC-3HA-Bla vector, and transfected into the bloodstream-form 221 cell line. Stable transfectants were selected under 10 µg/ml Blasticidin. To tag the endogenous TbTLK1 in the RNAi cell lines, pC-TbTLK1-3HA-Bla was transfected into the cells harboring the respective RNAi construct. Conversely, to tag the endogenous TbKIN-B, TbCPC1, TbCPC2, and TbAUK1 with triple HA in the TbTLK1 RNAi cell line, the individual linearized pC-3HA-Bla constructs were transfected into the TbTLK1 RNAi cells by electroporation. Stable transfectants were selected under 10 µg/ml Blasticidin in addition to Phleomycin, Hygromycin and G418. Correct *in situ* tagging of one of the two alleles was confirmed by PCR and subsequent sequencing in each case.

### Immunofluorescence Microscopy

The following primary antibodies were used: The anti-tyrosinated microtubule antibodies YL1/2 for staining the basal body [32], [33] (Chemicon, Temecula, CA); L8C4 for staining the flagellum [33]; KMX-1 for staining the spindle structure [33]; and FITC-conjugated anti-HA mAb (Sigma-Aldrich) for staining the HA-tagged proteins. For YL1/2 and L8C4 double staining, cells were fixed with cold methanol. For anti-HA immunostaining and anti-HA and KMX-1 double staining, cells were fixed with 4% paraformaldehyde. The fixed cells were incubated with the primary antibodies at room temperature for 60 min, washed three times and incubated with FITC-conjugated and Cy3-conjugated secondary antibodies (Sigma-Aldrich) for another 60 min at room temperature. After washing three more times, cells were stained with 1.0 µg/ml of 4, 6-diamino-2-phenylindole (DAPI) and the slides were mounted in Vectashield mount medium and examined under a fluorescence microscope.

### Yeast Two Hybrid Assay

Full-length TbTLK1, TbAUK1, TbKIN-A, TbKIN-B, TbCPC1 and TbCPC2 were each cloned into pGADT7 vector for expression of proteins fused to the Gal4 activation domain (prey) or into pGBKT7 vector for expression of proteins fused to the Gal4 binding domain (bait). Yeast strains AH109 (mating type a) and Y187 (mating type α) were transformed with prey or bait plasmids, respectively. Strains carrying different combinations of bait and prey were generated by mating the haploids in YPDA media for 24 hrs at 30°C, followed by plating on SD-Leu-Trp plates to select for the presence of both plasmids. Each combination strain was then spotted in four ten-fold serial dilutions onto SD-Leu-Trp and SD-His-Leu-Trp plates; the latter plate selects for clones carrying the bait protein and the prey protein that interact.

### Immunoprecipitation and Immunoblotting

*T. brucei* cells were incubated in lysis buffer (25 mM Tris-Cl, pH 7.6, 100 mM NaCl, 1% Nonidet P-40, 1 mM dithiothreitol, and protease inhibitor cocktail) for 30 min on ice and cleared by centrifugation. The cleared lysate was incubated with anti-HA pAb at 4°C for 1 h and precipitated with protein G Sepharose beads overnight. The immuno-precipitates thus collected were fractionated by SDS-PAGE, transferred onto PVDF membrane and immuno-blotted with anti-ProteinC mAb. Immuno-blotting was performed essentially as described previously [30].

## Supporting Information

Movie S1 Translocalization of TbCPC1-EYFP in live cell imaged by video fluorescence microscopy. Procyclic cell stably expressing the enhanced yellow fluorescent protein (EYFP) tagged TbCPC1 (green) was imaged during mitosis-cytokinesis. Time-lapse images of individual cells were acquired with a 6D High Throughput Microscope at the Nikon Imaging Center at UCSF (http://nic.ucsf.edu/6D.html). The images were taken at 5 to 8 multi-points with a fixed time interval (10 min). An auto-focusing program using DIC images was installed that produces images with Z-stacks at −1, 0 and +1 µm from the auto-focused plane. DNA was stained with Hoechst DNA dye (red), and the fluorescence and phase images were merged.
(0.64 MB MOV)
